# 

**Published:** 1875-01

**Authors:** 


### Contents of January Number.

### ORIGINAL DEPARTMENT.

ART. I.—The Nervous Accidents of Syphilis. By John B. Stonehouse, M. D., of Albany,.......... .... .............................. 201
ART. II.—Clinical Remarks upon Surgical Cases occurring in the Buffalo Hospital of the Sisters of Charity. By Prof. J. F. Miner, M. D. Reported by W. W. Miner, M. D. ................................. 226

### MISCELLANEOUS.

Prof. Erichsen and American Surgery,........... .................. 230

### EDITORIAL DEPARTMENT.

Erie County Medical Society,.......................................... 233
Homœopathic Physicians and Regular Medical Societies,............... 235
The State Legislature and Vivisection,........... .. ................ 236
Association of the Alumni and Officers Medical Department of the University of Buffalo,....... ...................................... 237
Items,..... ................ ................. .................. 239

BOOKS REVIEWED:

Essentials of the Principals and Practice of Medicine. By Henry Hartshorne, M. D.,........ ......................... 239
Books and Pamphlets Received .... .. ............................ 240

THE BUFFALO

